# Effect of type of diet on blood and plasma taurine concentrations, cardiac biomarkers, and echocardiograms in 4 dog breeds

**DOI:** 10.1111/jvim.16075

**Published:** 2021-02-27

**Authors:** Darcy Adin, Lisa Freeman, Rebecca Stepien, John E. Rush, Sonja Tjostheim, Heidi Kellihan, Michael Aherne, Michelle Vereb, Robert Goldberg

**Affiliations:** ^1^ University of Florida College of Veterinary Medicine Gainesville Florida USA; ^2^ Tufts University, Cummings School of Veterinary Medicine North Grafton Massachusetts USA; ^3^ University of Wisconsin School of Veterinary Medicine Madison Wisconsin USA; ^4^ University of Massachusetts Medical School Worcester Massachusetts USA

**Keywords:** dilated cardiomyopathy, food, grain free, grain inclusive, NT‐proBNP, troponin

## Abstract

**Background:**

Associations of diet with dilated cardiomyopathy are under investigation.

**Objectives:**

That cardiac assessment would show abnormalities in healthy dogs eating grain‐free (GF) diets or diets with Food and Drug Administration (FDA)‐listed ingredients of concern (peas, lentils, or potatoes) as top 10 ingredients (FDA‐PLP), but not in dogs eating grain‐inclusive (GI) diets or diets without FDA‐listed ingredients of concern (PLP) in the top 10 ingredients (NoFDA‐PLP).

**Animals:**

One hundred eighty‐eight healthy Doberman Pinschers, Golden Retrievers, Miniature Schnauzers, and Whippets.

**Methods:**

This study was an observational cross‐sectional study. Echocardiograms, cardiac biomarkers, and blood and plasma taurine concentrations were compared between dogs eating GF (n = 26) and GI (n = 162) diets, and between FDA‐PLP (n = 39) and NoFDA‐PLP (n = 149) diets, controlling for age and breed. Demographic characteristics, murmurs, genetic status, and ventricular premature complexes (VPCs) during examination were compared between dogs eating different diet types.

**Results:**

No differences in echocardiographic variables, N‐terminal pro‐B‐type natriuretic peptide or whole blood taurine were noted between dogs eating different diet types. Dogs eating GF diets had higher median high‐sensitivity cardiac troponin I (hs‐cTnI) (GF 0.076 ng/mL [Interquartile range (IQR), 0.028‐0.156] vs. GI 0.048 [IQR, 0.0026‐0.080]; *P* < .001) and higher median plasma taurine (GF 125 nmol/mL [IQR, 101‐148] vs GI 104 [IQR, 86‐123]; *P* = .02) than dogs eating GI diets. Dogs eating FDA‐PLP diets had higher median hs‐cTnI (0.059 ng/mL [IQR, 0.028‐0.122]) than dogs eating NoFDA‐PLP diets (0.048 [IQR, 0.025‐0.085]; *P* = .006). A greater proportion of dogs eating FDA‐PLP diets (10%) had VPCs than dogs eating NoFDA‐PLP diets (2%; *P* = .04*)*.

**Conclusions and Clinical Importance:**

Higher hs‐cTnI in healthy dogs eating GF and FDA‐PLP diets might indicate low‐level cardiomyocyte injury.

**Abbreviations**
EF: ejection fraction
FDA: Food and Drug Administration
FDA‐PLP: diets with FDA‐listed ingredients of concern (peas, lentils, potatoes) in the top 10 ingredients
FS: fractional shortening
GF: grain free
GI: grain inclusive
hs‐cTnI: high‐sensitivity cardiac troponin I
LA/Ao: left atrial to aortic diameter
LV: left ventricular
LVIDdN: normalized left ventricular internal diameter in diastole
LVIDsN: normalized left ventricular internal diameter in systole
LVVd/m^2^: left ventricular volume in diastole indexed to body surface area
LVVs/m^2^: left ventricular volume in systole indexed to body surface area
nDCM: nutritional dilated cardiomyopathy
NoFDA‐PLP: diets without FDA‐listed ingredients of concern (peas, lentils, potatoes) in the top 10 ingredients
NT‐proBNP: N‐terminal pro B‐type natiuretic peptide
PLP: peas, lentils, potatoes

## 1 INTRODUCTION

Dilated cardiomyopathy is a disease of dogs that is associated with short duration of survival once clinical signs of congestive heart failure are detected.^1^, ^2^ The disease has genetic causes in several large and giant breeds and is uncommon in small breed dogs.^1^, ^3^, ^4^, ^5^ The increased recognition of dilated cardiomyopathy in atypical breeds in recent years, coupled with the observation of dietary commonalities in these dogs, prompted clinical publications and increased reporting of affected cases to the Food and Drug Administration (FDA).^6^, ^7^, ^8^ Reports describing dogs suspected to have a nutritional basis for this cardiac condition, and recovery of some dogs after nutritional intervention are currently limited to a small retrospective study^6^ and 2 prospective studies.^7^, ^8^ Dietary characteristics possibly associated with nutritional dilated cardiomyopathy (nDCM) include the absence of grains and inclusion of peas, lentils, or potatoes as main ingredients. The accurate classification and description of diets implicated in nDCM has been elusive.

Theoretical reasons for dog foods that are grain free (GF) or high in peas, lentils, or potatoes to be associated with nDCM include nutritional deficiencies, poor nutrient bioavailability, impaired taurine metabolism or precursor availability, nutrient‐to‐nutrient interactions, or presence of toxic substances.^9^ Genetic makeup or other modifying factors could also influence disease expression in some dogs and explain why not all dogs eating these diets are affected. Alternatively, it remains possible that this apparent association is confounded by other factors.^10^ Although some affected dogs are taurine deficient (especially Golden Retrievers^7^), thereby strengthening the association with food, the majority of dogs with suspected nDCM have normal blood taurine concentrations.^6^ The role of taurine is uncertain at this time.^11^, ^12^, ^13^, ^14^

Nutritional dilated cardiomyopathy is a complex disorder with uncertain etiologies, undetermined contribution of dietary factors, variable phenotype, and unclear extradietary modifiers of disease expression. Many diseases have a spectrum of severity ranging from mild to overt disease, and so it is possible that some healthy dogs have subclinical cardiac abnormalities related to diet type. One study reported echocardiograms from clinically healthy Golden Retrievers,^8^ but additional studies evaluating for subclinical disease related to diet are needed to facilitate efforts at early intervention and to enhance understanding of the natural history and pathophysiology of nDCM. The purpose of this observational study was to evaluate the effect of diet type on blood and plasma taurine concentrations, cardiac biomarkers, and echocardiograms in apparently healthy dogs of 4 breeds. We hypothesized that echocardiographic chamber dimensions and cardiac biomarkers would be higher in dogs eating diets that are GF or high in peas, lentils, or potatoes, compared with dogs eating diets without these characteristics, and that taurine concentrations would not differ between dogs eating diets with and without these characteristics.

## 2 METHODS

This study was an observational cross‐sectional study that was approved by the Institutional Animal Care and Use Committees at the University of Florida, College of Veterinary Medicine (#201810504) and the University of Wisconsin, School of Veterinary Medicine (#V005029‐R01). All dog owners provided informed consent. Dogs were recruited for study enrollment at the University of Florida and the 2019 American Whippet Club National Specialty breed show in Topeka, Kansas. Dogs were recruited and enrolled irrespective of age, sex, or diet type.

### 2.1 Inclusion criteria

Dogs were included if they were considered to be clinically healthy, had been eating a commercial dog food for at least 6 months, and were a purebred Whippet, Golden Retriever, Doberman Pinscher, or Miniature Schnauzer. We specifically selected 2 breeds with no known genetic predisposition to dilated cardiomyopathy (Miniature Schnauzer and Whippet), 1 with known heritable dilated cardiomyopathy (Doberman Pinscher), and 1 that appears to be at increased risk for nDCM (Golden Retriever), although appropriate epidemiological data for this condition are lacking. Four breeds with notably different characteristics were chosen for this study to test the hypotheses in diverse breeds without introducing extensive breed variability. The study was not designed to evaluate each breed individually.

### 2.2 Exclusion criteria

Dogs were not included if they were fed more than 1 type of food within the 6 months before presentation, were receiving taurine supplementation, were receiving cardiac medications, or had known cardiac disease. Dogs with clinically important extracardiac diseases were excluded, as were female dogs that were pregnant, lactating, or in estrous.

### 2.3 Procedures

#### 2.3.1 Examination and diet history

Body weight, body condition score (1‐9 scale),^15^ muscle condition score (normal, mild, moderate, or severe muscle loss),^16^ and results of a physical examination were recorded for all dogs. Owners filled out a diet history form detailing specific foods, treats, supplements, and the duration of feeding. Biotin containing supplements were specifically noted because of the potential for mega‐doses of this vitamin to interfere with high‐sensitivity cardiac troponin I (hs‐cTnI) measurement.^17^, ^18^

#### 2.3.2 Categorization of diets

The ingredient list of the diets fed to enrolled dogs were reviewed for categorization based on the presence of grains and separate categorization based on whether peas, lentils, or potatoes were listed in the top 10 ingredients based on the FDA's advisory (https://www.fda.gov/animal‐veterinary/outbreaks‐and‐advisories/fda‐investigation‐potential‐link‐between‐certain‐diets‐and‐canine‐dilated‐cardiomyopathy). Diets were categorized as GF if there were no grain‐containing or grain‐derived ingredients listed and as grain inclusive (GI) if grain‐containing or grain‐derived ingredients were listed. Grain‐containing or grain‐derived ingredients included whole‐grain products or refined‐grain products made from any part of wheat, rice, oats, corn, barley, or another cereal grain.^19^ Oils were not considered a grain product. Diets were separately categorized as having FDA‐listed ingredients of concern (peas, lentils, or potatoes) in the top 10 ingredients (FDA‐PLP) or as without FDA‐listed ingredients of concern (peas, lentils, or potatoes) in the top 10 ingredients (NoFDA‐PLP). Inclusion of taurine and methionine as dietary ingredients was noted because these amino acids could affect blood taurine concentrations.^20^

#### 2.3.3 Echocardiography

A standard echocardiogram was performed without sedation and with monitoring electrocardiography. Echocardiographic variables assessing left ventricular (LV) size and function were measured offline after all dogs were enrolled, with the investigators blinded to dog identity and dietary history. Reported values are the average of triplicate measurements. Two‐dimensional volume indices were determined using a 4‐chamber, right‐sided long‐axis view and the modified disk summation method.^21^ Left ventricular volumes in diastole and systole were indexed to body surface area (LVVd/m^2^ and LVVs/m^2^, respectively).^22^ Ejection fraction (EF) was calculated as the percent change in LV volume between diastole and systole. The right‐sided short‐axis view at the level of the chordae tendineae was used for M‐mode measurements of LV size including LV internal dimension in diastole and systole normalized for body length using allometric scaling according to 2 formulas (normalized LV internal diameter in diastole [LVIDdN] and normalized LV internal diameter in systole [LVIDsN], respectively).^23^, ^24^ Fractional shortening (FS) was calculated as the percent change in LV diameter between diastole and systole using M‐mode measurements. Left atrium to aortic diameter ratio (LA/Ao) was determined in the right‐sided short‐axis parasternal view in mid‐diastole using 2D measurements.^25^ Rhythm during the echocardiogram was noted.

#### 2.3.4 Genotyping

Doberman Pinschers were tested at the North Carolina State University Veterinary Genetics Laboratory (Raleigh, North Carolina) using a buccal swab for genetic mutations (DCM1 and DCM2) associated with dilated cardiomyopathy in this breed.^3^, ^4^

#### 2.3.5 Blood sampling

Blood was collected by peripheral venipuncture into either 3‐mL tubes (Doberman Pinchers, Whippets, Golden Retrievers) or 1‐mL tubes (Miniature Schnauzers) for subsequent testing. Only nonhemolyzed serum and plasma samples were used for testing. Lithium heparinized whole blood and plasma samples were analyzed for whole blood and plasma taurine concentrations by the Amino Acid Laboratory, University of California, Davis (Davis, California). Serum samples were analyzed for hs‐cTnI concentrations by the Gastrointestinal Laboratory, Texas A & M University (College Station, Texas) using the ADVIA Centaur Troponin I Ultra analyzer (Siemens Healthcare Diagnostics, Deerfield, Illinois) which has been validated for use in dogs.^26^ Ethylenediaminetetraacetic acid (EDTA) plasma samples were analyzed for N‐terminal pro B‐type natriuretic peptide (NT‐proBNP) concentrations by IDEXX Laboratories (Westbrook, Maine). In‐house SNAP 4Dx test (IDEXX Laboratories, Westbrook, Maine) was performed on EDTA plasma of dogs enrolled at the University of Florida that had elevated hs‐cTnI concentrations to screen for common infectious diseases that could cause cardiac troponin I elevations.

### 2.4 Statistical analysis

Commercial software was used for data analysis (SAS 9.4, Cary, North Carolina). Data are presented as median and interquartile range (IQR). Differences in the baseline characteristics of dogs in each diet type group were compared using Fisher's exact test or chi‐square test and Mann‐Whitney test for categorical and continuous variables, respectively. Differences in selected echocardiographic variables (LVIDdN and LVIDsN using both allometric formulas, LA/Ao, FS, LVVD/m^2^, LVVS/m^2^, EF), whole blood and plasma taurine concentrations, hs‐cTnI concentrations, and NT‐proBNP concentrations were compared between dogs eating GF and GI diets and between dogs eating FDA‐PLP and NoFDA‐PLP diets using analysis of variance tests. Crude, unadjusted analyses were performed to examine the association between the different diet categorizations and our principal study outcomes. We then performed multivariable regression analyses examining these associations while controlling for the dogs' age and breed, yielding multivariable adjusted *P* values.

The prevalence of murmurs, occurrence of arrhythmias noted on monitoring electrocardiogram during echocardiography, and genetic positivity for Doberman Pinschers (heterozygous or homozygous for either mutation) were compared between dogs eating GF and GI diets, and between dogs eating FDA‐PLP and NoFDA‐PLP diets using Fisher's exact test. Significance was set at *P* < .05.

## 3 RESULTS

### 3.1 Study population

A total of 188 dogs met the inclusion criteria and were enrolled in this study. All enrolled Whippets (n = 86) were evaluated over a 3‐day period at the 2019 American Whippet Club National Specialty breed show in Topeka, Kansas. Dogs from the other 3 breeds (37 Doberman Pinschers, 43 Golden Retrievers, and 22 Miniature Schnauzers) were enrolled at the University of Florida between May 2019 and July 2020.

There were no significant differences in terms of age, weight, sex, or time eating the diet before enrollment according to the type of diet consumed (Table 1). There were no differences in genetic status between Doberman Pinschers fed GF and GI diets or between Doberman Pinschers fed FDA‐PLP and NoFDA‐PLP diets. No differences in murmur prevalence were present between diet types. All murmurs were low grade (1/6 or 2/6). The proportion of dogs with ventricular premature complexes (VPCs) noted on the monitoring electrocardiogram during echocardiography was significantly higher for dogs eating FDA‐PLP diets compared with NoFDA‐PLP diets (*P* = .04).

**TABLE 1:** Demographic characteristics, genetic status, murmurs, and ventricular premature complexes in dogs based on diet characterization

	GF (n = 26)	GI (n = 162)	*P* value	FDA‐PLP (n = 39)	NoFDA‐PLP (n = 149)	*P* value
Age (months)	50 (36‐99)	49 (30‐79)	.38	45 (24‐87)	50 (30‐81)	.99
Weight (kg)	24.3 (9.1‐32.9)	17.7 (14.2‐29.6)	.87	24.0 (13.4‐32.0)	17.7 (14.2‐29.4)	.75
Months on diet before presentation	20 (15‐48)	24 (12‐40)	.48	21 (15‐47)	24 (11‐40)	.37
Male	10 (39%)	61 (38%)	1.0	11 (28%)	60 (40%)	.20
Positive genetic status DP (n = 37)	5 (71%)	18 (60%)	.69	5 (56%)	18 (64%)	.70
Murmur	6 (23%)	46 (28%)	.70	11 (28%)	41 (28%)	.65
VPCs during echocardiogram (# of each breed)	3 (12%) (2 DP, 1 GR)	4 (3%) (3 DP, 1 GR)	.06	4 (10%) (2 DP, 2 GR)	3 (2%) (3 DP)	**.04**

*Note*: Median and interquartile range are shown for the first 3 variables. Number and percentage of the total are shown for the remaining variables. Genetic status was considered positive if Doberman Pinschers were heterozygous or homozygous for either the DCM1 or DCM2 mutations. *P* < .05 was considered significant (bolded).

Abbreviations: DP, Doberman Pinschers; FDA‐PLP, diets with Food and Drug Administration ingredients of concern (peas, lentils, or potatoes) in the top 10 ingredients; GF, grain‐free diets; GI, grain‐inclusive diets; GR, Golden Retrievers; NoFDA‐PLP, diets without Food and Drug Administration ingredients of concern (peas, lentils, or potatoes) in the top 10 ingredients; VPCs, ventricular premature complexes.

The majority (92%) of dogs in the study were assessed as has having normal muscle condition. Muscle condition scores were not significantly different between diet categorizations after controlling for age and breed (median and IQR scores were normal for all groups; adjusted *P* = .70 comparing GF to GI and adjusted *P* = .63 comparing FDA‐PLP to NoFDA‐PLP). Median [IQR] body condition score was 5.0 [5.0‐6.0] for dogs eating GF and FDA‐PLP diets and 5.0 [5.0‐5.0] for dogs eating GI and NoFDA‐PLP diets. Body condition scores were not different between diet categorizations after controlling for age and breed (adjusted *P* = .76 for GF compared to GI and adjusted *P* = 0.10 for FDA‐PLP compared to NoFDA‐PLP).

### 3.2 Diets and supplements

The 188 dogs in this study were fed 72 diets, which were represented by 34 different brands. All diets were dry kibble. Table 2 shows the number of dogs from each breed that were eating each diet type.

**TABLE 2:** Breed distribution for each diet type classification

	GF (n = 26)	GI (n = 162)	FDA‐PLP (n = 39)	NoFDA‐PLP (n = 149)
Doberman Pinscher	7	30	9	28
Golden Retriever	8	35	12	31
Miniature Schnauzer	8	14	8	14
Whippet	3	83	10	76

Abbreviations: FDA‐PLP, diets with Food and Drug Administration ingredients of concern (peas, lentils, or potatoes) in the top 10 ingredients; GF, grain‐free diets; GI, grain‐inclusive diets; NoFDA‐PLP, diets without Food and Drug Administration ingredients of concern (peas, lentils, or potatoes) in the top 10 ingredients.

#### 3.2.1 Categorization based on GI

There were 21 GF diets (15 different manufacturers) and 51 GI diets (22 different manufacturers) (Table S1). The proportion of diets with taurine or methionine included as ingredients was not statistically different between diet types (GF 16/21; 76%, GI 28/51; 55%, *P* = .12). Twenty‐six dogs were eating GF diets and 162 dogs were eating GI diets. The proportion of dogs eating GF diets that included taurine or methionine as ingredients (21/26; 81%) was greater than the proportion of dogs eating GI diets that included taurine or methionine as ingredients (63/162; 39%, *P* < .001).

#### 3.2.2 Categorization based on FDA ingredients of concern in top 10 ingredients

There were 29 FDA‐PLP diets (21 different manufacturers) and 43 NoFDA‐PLP diets (19 different manufacturers) (Table S2). The proportion of diets with taurine or methionine included as ingredients was not statistically different between diet types (FDA‐PLP 22/29; 76%, NoFDA‐PLP 22/43; 51%, *P* = 0.05). Thirty‐nine dogs were eating FDA‐PLP diets and 149 dogs were eating NoFDA‐PLP diets. The proportion of dogs eating FDA‐PLP diets that included taurine or methionine as ingredients (29/39; 74%) was greater than the proportion of dogs eating NoFDA‐PLP diets that included taurine or methionine as ingredients (55/149; 37%, *P* < .001).

#### 3.2.3 Biotin supplementation

A minority (3.2%; 6/188) of dogs were receiving biotin supplementation as a component of a multivitamin fed separate from their main diet. The proportion of dogs eating GF diets that received biotin supplementation (1/26; 3.8%) was not different from the proportion of dogs eating GI diets that received biotin supplementation (5/162; 3.1%, *P* = 1.0). Findings were similar for dogs eating FDA‐PLP diets (1/39; 2.5% received biotin supplementation) and NoFDA‐PLP diets (5/149; 3.4% received biotin supplementation; *P* = 1.0).

### 3.3 Echocardiography, cardiac biomarkers, and taurine concentrations

After multivariable regression, no differences were found between diet categorizations (GF vs GI and FDA‐PLP vs NoFDA‐PLP) for any echocardiographic variables, NT‐proBNP, or whole blood taurine concentrations (Table 3). Median hs‐cTnI (*P* < .001) and median plasma taurine concentrations (*P* = .02) were both significantly higher in dogs eating GF diets compared to GI diets and median hs‐cTnI concentration (*P* = .006) was significantly higher in dogs eating FDA‐PLP diets compared to NoFDA‐PLP diets (Table 3).

**TABLE 3:** Differences between diet categorizations for echocardiographic variables, taurine concentrations, and cardiac biomarkers

	GF (n = 26)	GI (n = 162)	Unadjusted *P* value	Adjusted *P* value	FDA‐PLP (n = 39)	NoFDA‐PLP (n = 149)	Unadjusted *P* value	Adjusted *P* value
LVIDdN (C)	1.50 (1.43‐1.70)	1.62 (1.49‐1.75)	.11	.23	1.51 (1.42‐1.73)	1.62 (1.50‐1.74)	.07	.99
LVIDdN (V)	1.49 (1.40‐1.67)	1.59 (1.47‐1.72)	.11	.23	1.49 (1.39‐1.70)	1.60 (1.47–1.72)	.07	.99
LVIDsN (C)	.95 (.85‐1.05)	1.07 (.96‐1.19)	.02	.34	1.00 (.86‐1.10)	1.07 (.96‐1.19)	.03	.89
LVIDsN (V)	.78 (.68–.85)	.85 (.74–.97)	.02	.25	.81 (69–.88)	.85 (.74–.97)	.04	.96
FS (%)	34.0 (28.5‐38.3)	28.9 (24.7‐34.3)	.03	.59	33.9 (26.5‐38.1)	28.9 (25.0‐33.7)	.17	.75
LA/Ao	1.31 (1.25‐1.38)	1.35 (1.25‐1.45)	.22	.67	1.33 (1.25‐1.39)	1.35 (1.26‐1.45)	.28	.58
LVVd/m^2^	60.0 (44.3‐75.0)	76.1 (55.8‐95.7)	.004	.19	59.8 (51.4‐79.0)	76.9 (56.9‐95.1)	.01	.85
LVVs/m^2^	24.9 (15.6‐31.7)	31.2 (21.5‐41.2)	.05	.18	24.5 (16.5‐36.0)	31.4 (21.6‐41.2)	.03	.99
EF (%)	60.0 (55.1‐64.6)	58.9 (54.0‐65.0)	.90	.21	60.7 (55.1‐65.3)	58.5 (53.2‐64.0)	.43	.98
hs‐cTnI (ng/mL)	.076 (.028–.156)	.048 (.026‐.080)	.001	**<.001**	.059 (.028–.122)	.048 (.025‐.085)	.03	**.006**
NT‐proBNP (pmol/L)	429 (250‐653)	358 (250‐586)	.77	.85	414 (250‐747)	357 (250‐562)	.63	.97
Whole blood taurine (nmol/mL)	281 (243‐316)	227 (194‐278)	.002	.99	268 (220‐303)	228 (193‐282)	.05	.77
Plasma taurine (nmol/mL)	125 (101–148)	104 (86‐123)	<.001	**.02**	121 (94‐142)	104 (86‐121)	.03	.10

*Note*: Median and interquartile range are shown with crude, unadjusted *P* values obtained from the univariable analyses and adjusted *P* values after multivariable regression analysis controlling for age and breed. *P* < .05 after multivariable adjustment was considered to be statistically significant (bolded).

Abbreviations: EF, ejection fraction; FDA‐PLP, diets with Food and Drug Administration ingredients of concern (peas, lentils, or potatoes) in the top 10 ingredients; GF, grain‐free diets; GI, grain‐inclusive diets; hs‐cTnI, high‐sensitivity cardiac troponin I; LVIDdN (C), normalized left ventricular internal diameter in diastole by Cornell et al. formula^23^; LVIDdN (V), normalized left ventricular internal diameter in diastole by Visser et al formula^24^; LVIDsN (C), normalized left ventricular internal diameter in systole by Cornell et al. formula^23^; LVIDsN (V), normalized left ventricular internal diameter in systole by Visser et al. formula^24^; FS, fractional shortening; LA/Ao, left atrial to aortic ratio; LVVd/m^2^, left ventricular volume in diastole indexed to body surface area; LVVs/m^2^, left ventricular volume in systole indexed to body surface area; NoFDA‐PLP, diets without Food and Drug Administration ingredients of concern (peas, lentils, or potatoes) in the top 10 ingredients; NT‐proBNP, N‐terminal pro B‐type natriuretic peptide.

### 3.4 SNAP 4Dx

Twenty‐eight dogs enrolled at the University of Florida showed elevated serum hs‐cTnI concentrations and so underwent in‐house SNAP 4Dx testing. All dogs were negative for *Dirofilaria immitis* antigen, *Anaplasma phagocytophilum* antibody, *Anaplasma platys* antibody, *Ehrilichia canis* antibody, and *Ehrlichia ewingii* antibody. One dog was determined to be positive for *Borrelia burgdoferi* C6 antibody. This dog was eating a diet categorized as GI and NoFDA‐PLP.

## 4 DISCUSSION

This study of 4 dog breeds found significantly higher hs‐cTnI concentrations in healthy dogs eating GF diets compared to dogs eating GI diets, and in healthy dogs eating FDA‐PLP diets compared to dogs eating NoFDA‐PLP diets, and these differences persisted after controlling for the dogs' age and breed. Serum concentrations of cardiac troponin I are low in healthy dogs.^27^ Myocardial cell injury is associated with release of troponin I into the circulation, allowing for its detection by analyzers.^27^, ^28^ Low‐level increases in serum cardiac troponin I concentrations occur in many cardiac diseases including in people with unhealthy dietary profiles.^27^, ^29^, ^30^ Although biotin supplementation (but not dietary biotin) can interfere with hs‐cTnI by many analyzers,^17^, ^18^ this possible concern most likely did not influence the current results because only a small minority of dogs received biotin‐containing dietary supplements, with equal proportions of dogs between diet types. The importance of low‐level increases in serum cardiac troponin I are not always clear, but in people are associated with both exercise and subclinical cardiac injury.^29^, ^30^, ^31^ The hs‐cTnI has prognostic value in dogs with degenerative mitral valve disease, with concentrations above a relatively low cutoff of .025 ng/mL associated with decreased survival.^32^ In addition, low‐level cardiac troponin T concentrations induced in rats by monosodium glutamate administration correlates with other blood markers of cardiac injury and histologic evidence of cardiac myofiber swelling and degeneration.^33^ The reasons for the higher hs‐cTnI concentrations in dogs eating GF and FDA‐PLP diets could not be determined from this study, but this finding provides basic mechanistic information to guide future studies of the etiology of nDCM.

Dogs eating GF diets as a group also had higher plasma, but not whole blood taurine concentrations compared to dogs eating GI diets. Differences in blood or plasma taurine concentrations were not found for dogs eating FDA‐PLP and NoFDA‐PLP diets. The higher plasma taurine concentration in dogs eating GF diets compared to dogs eating GI diets could be a result of the inclusion of taurine or methionine as ingredients in the diets eaten by more dogs in the GF group compared to the GI group. Oral supplementation with taurine and dietary inclusion of taurine and methionine increase plasma taurine concentrations independent of whole blood taurine concentrations^34^, ^35^ but 1 study showed both higher whole blood and plasma taurine concentrations in dogs eating diets fortified with taurine and methionine.^20^ Alternatively, the higher plasma taurine concentrations in dogs eating GF diets might be caused by cardiomyocyte injury, which could be supported by the finding that hs‐cTnI was also higher in dogs eating GF diets compared to GI diets. Circulating taurine concentrations have historically been used as a biomarker of myocardial injury in people, although whole blood concentrations seem more closely associated with injury than plasma concentrations in some studies.^36^, ^37^, ^38^ Elevated plasma taurine concentrations in conjunction with low intramyocardial taurine concentrations in a rat model of isoproterenol‐induced myocardial injury resulted from defective myocardial taurine transport and increased sarcolemmal taurine release associated with cardiomyocyte damage.^39^ Taurine supplementation to rats increases expression of the myocardial taurine transporter, restoring taurine movement into the myocardial cells.^39^ Our results contrast with a study of healthy Golden Retrievers, which found no difference in plasma taurine concentrations, but lower whole blood taurine concentrations in dogs eating non‐traditional diets compared to traditional diets.^8^ The reasons for these contrasting findings are unclear, but might be influenced by the other, diverse breeds included in our study, different diets that were fed to dogs in each study, and different diet characterizations used in each study. While the reasons that dogs consuming GF diets had higher plasma taurine concentrations in our study are unknown, this finding could be related to dietary inclusion of taurine or methionine as an ingredient, sample handling artifacts, or myocardial disease and injury.

Although the number of dogs with VPCs noted on monitoring electrocardiography during the echocardiogram was relatively low, a greater proportion of dogs eating FDA‐PLP diets had VPCs than those eating NoFDA‐PLP diets. No echocardiographic differences were found between healthy dogs in this study eating GF and GI diets or between healthy dogs in this study eating FDA‐PLP and NoFDA‐PLP diets. In addition, no diet type differences were detected for NT‐proBNP. The lack of differences in echocardiographic measurements is in contrast to a recent study in Golden Retrievers in which FS was lower and LV diameters were larger in those eating nontraditional diets compared to traditional diets.^8^ Similar to postulated reasons for contrasting findings regarding taurine concentrations in that publication, our study enrolled 3 breeds in addition to Golden Retrievers and utilized different diet categorizations. The absence of echocardiographic differences between dogs eating different diet types in the current study could be because these dogs were clinically healthy with a low disease prevalence, breed differences and variability, low statistical power, or to true absence of differences. We intentionally chose to study 2 breeds that are not predisposed to dilated cardiomyopathy (Miniature Schnauzer and Whippet), 1 breed that is predisposed to genetically‐based dilated cardiomyopathy (Doberman Pinscher), and 1 breed that appears predisposed to nDCM and taurine deficiency (Golden Retriever). The unique differences between the studied breeds might have reduced our ability to detect small differences between groups^40^, ^41^ and our study was not planned or powered to evaluate each breed individually. However, we consider that the subtle, but statistically significantly higher concentrations of hs‐cTnI in these clinically healthy dogs eating GF and FDA‐PLP diets could represent subclinical cardiac injury that would not be expected to be associated with gross echocardiographic changes. This study could not determine whether the higher hs‐cTnI in these dogs has potential to become clinically important through eventual progression over time to echocardiographic changes and overt cardiac disease, or whether this finding is even clinically important for these dogs.

The correct characterization of diets that are associated with nDCM is unknown because the cause has not yet been identified. We chose to group diets by 2 categorizations, based on the absence or presence of grains and based on the absence or presence of FDA‐listed ingredients of concern in the top 10 ingredients. Although these 2 categorizations are similar, there were some diets that were included in 1 categorization but not another because of specific ingredients used and the ingredient list position. For example, some GF diets listed peas, lentils, or potatoes after the 10th ingredient and some GI diets had peas, lentils, or potatoes in the top 10 ingredients. It should be noted that it is impossible to determine the exact amount of an ingredient based on the ingredient list. Accurate description of diets of concern remains elusive but in light of the hs‐cTnI differences detected in this study, dietary commonalities between GF and FDA‐PLP diets might prove useful for designing future studies. This study was not intended to implicate or exonerate specific diets. The differences between the 2 ways diets were categorized, the many different diets and varieties on the market (many of which were not fed to dogs in this study), the small number of dogs eating specific diets in this study, and the variety of ingredients included in different diets, limit the ability to make specific dietary recommendations based on the results of this study. Likewise, the diet categorizations explored in this study should not be viewed as absolute: for example, the use of peas, lentils, or potatoes in the top 10 ingredients is observational and the cutoff at the 10th ingredient is somewhat arbitrary; this does not mean that these ingredients below this level are not of concern. The results of this study, however, do support the finding that diets fed to clinically healthy dogs of these 4 breeds for at least 6 months that are GF or contain peas, lentils, or potatoes as top 10 ingredients are associated with higher hs‐cTnI concentrations compared to diets without these characteristics.

This study has several important limitations to be considered during interpretation and application of findings. In an attempt to limit breed variability while evaluating several kinds of dogs, we chose to evaluate only 4 breeds. As such, the results of this study are only applicable to Doberman Pinschers, Golden Retrievers, Miniature Schnauzers, and Whippets. The statistical power might have been too low to detect differences between diet types for some variables, especially considering the unbalanced group sizes. In addition, the study was not designed to analyze each breed separately, and as such, the relatively small number of dogs in each breed prevented breed‐specific analyses. Based on other published studies, we presume that the higher hs‐cTnI in dogs eating GF and FDA‐PLP diets indicates low‐level cardiomyocyte injury^27^, ^28^, ^29^, ^30^; but this hypothesis is unproven and there could be other reasons for the observed differences. While we screened dogs with hs‐cTnI elevations from the University of Florida for some infectious diseases, we did not rule out all causes of myocarditis in this group of dogs. Lastly, the true prevalence of various cardiac arrhythmias in each diet type was not evaluated in this study because 24‐hour Holter monitoring was not performed.

## CONFLICT OF INTEREST DECLARATION

Dr Adin acknowledges research support from Nestle Purina PetCare and is a consultant and sponsored lecturer for Ceva Animal Health and Boehringer Ingelheim. Dr Freeman has received funding from, given sponsored lectures for, or provided professional services to Aratana Therapeutics, Elanco, Hill's Pet Nutrition, Nestlé Purina PetCare, P&G Petcare (now Mars), and Royal Canin. Dr Stepien is a consultant for Nestle Purina Petcare and has served as consultant or sponsored lecturer for IDEXX, Ceva Animal Health, and Boehringer Ingelheim. Dr Rush has received funding from, given sponsored lectures for, or provided professional services to Aratana Therapeutics, Elanco, Hill's Pet Nutrition, Nestlé Purina PetCare, Royal Canin, IDEXX and Boehringer Ingelheim. Dr Tjostheim does not report conflicts of interest. Dr. Kellihan does not report conflicts of interest. Dr Aherne does not report conflicts of interest. Ms Vereb does not report conflicts of interest. Dr Goldberg does not report conflicts of interest. The conflicts of interest reported by the authors did not influence data collection, data interpretation, or manuscript preparation.

## OFF‐LABEL ANTIMICROBIAL DECLARATION

No off‐label antimicrobial use.

## INSTITUTIONAL ANIMAL CARE AND USE COMMITTEE (IACUC) OR OTHER APPROVAL DECLARATION

This study was approved by the Institutional Care and Use Committees at the University of Florida, College of Veterinary Medicine (#201810504) and the University of Wisconsin, School of Veterinary Medicine (#V005029‐R01).

## HUMAN ETHICS APPROVAL DECLARATION

Authors declare human ethics approval was not needed for this study.

## Supporting information


**TABLE S1** Grain‐free and grain‐inclusive diets that were fed to enrolled dogs

**TABLE S2** Diets with FDA‐listed ingredients of concern (peas, lentils, or potatoes) in the top 10 ingredients (FDA‐PLP) and diets without FDA‐listed ingredients of concern in the top 10 ingredients (NoFDA‐PLP) that were fed to enrolled dogs
