# Supplementary material for: Effect of type of diet on blood and plasma taurine concentrations, cardiac biomarkers, and echocardiograms in 4 dog breeds
Source: J Vet Intern Med. 2021 Feb 27;35(2):771–9. doi: 10.1111/jvim.16075 (PMC7995416; doi:10.1111/jvim.16075)
Supplement: Supplementary file 1 — TABLE S1 Grain‐free and grain‐inclusive diets that were fed to enrolled dogs [file JVIM-35-771-s002.pdf]

Table S1. Grain-free and grain-inclusive diets that were fed to enrolled dogs.

**Grain-free diets**	**Grain-inclusive diets**
4Health Grain Free Chicken & Vegetables Formula Adult	4health Original Chicken & Rice Formula Adult
Acana Heritage Free-Run Poultry Formula	4health Original Performance
Acana Light and Fit Formula	4health Original Small Bites Formula Adult
Acana Meadowland	Actr1um Holistic
Aldi Pure Being Grain Free Salmon and Sweet Potato	American Journey Active Life Formula Large Breed Chicken, Brown Rice and Vegetables
Earthborn Holistic Primitive Natural	Avoderm Adult Chicken Meal & Brown Rice Formula
Fromm Salmon Tunalini Recipe	Bil-Jac Adult Select Formula
Honest Kitchen Zeal Grain Free Fish Recipe Dehydrated	Diamond Naturals All Life Stages Dog Chicken & Rice Formula
Merrick Grain Free Real Duck and Sweet Potato Recipe	Eukanuba Small Bites Chicken dry
Merrick Grain Free Real Lamb and Sweet Potato Recipe	Evanger's Chicken and Brown Rice Recipe
Natures Recipe Grain Free Chicken, Sweet Potato, & Pumpkin Recipe	Farmina N & D Ancestral Grain Chicken & Pomegranate Medium & Maxi Adult Dry
Nutrisource Pure Vita Salmon & Peas Entrée	Fromm Adult Gold Dry
Orijen Original Adult	Fromm Chicken A La Veg Recipe
Redford Naturals Limited Ingredient Diet Grain Free Large Breed Lamb & Sweet Potato Recipe Adult	Fromm Duck A La Veg Recipe
Simply Nourish Limited Ingredient Diet Salmon & Sweet Potato Recipe	Fromm Pork & Applesauce Formula
Taste of the Wild High Prairie Canine Recipe with Roasted Bison and Roasted Venison	Halo Holistic Wild Salmon and Whitefish Recipe Adult
Taste of the Wild Southwest Canyon Canine Recipe with Wild Boar	Hills Science Diet Digestive Care i/d Low Fat
Taste of the Wild Pacific Stream Canine Recipe with Smoked Salmon	Hill's Science Diet Digestive Care i/d
Taste of the Wild Pine Forest Canine Recipe with Venison & Legumes	Iams ProActive Health Adult Minichunks
Wellness Core Grain Free	Instinct Be Natural Real Chicken & Brown Rice Recipe
Zignature Salmon Limited Ingredient Formula	Kirkland Signature Adult Formula Chicken, Rice, & Vegetable
	Nutrisource Adult Chicken & Rice Recipe
	Nutro Wholesome Essentials Large Breed Adult Pasture-Fed Lamb & Rice Recipe

	Nutro Ultra Chicken/Lamb/Salmon Small Breed Weight Management
	Purina Beneful Healthy Puppy Dry Dog Food with Farm-Raised Chicken
	Purina One Smartblend Digestive Health Formula with Real Chicken
	Purina One Smartblend Chicken & Rice Formula
	Purina One Smartblend Lamb & Rice Formula
	Purina Pro Plan Savor Shredded Adult Beef & Rice Formula
	Purina Pro Plan Bright Mind Chicken & Rice Formula
	Purina Pro Plan Savor Shredded Blend Chicken and Rice Savor Formula
	Purina Pro Plan Focus Large Breed Chicken & Rice Formula
	Purina Pro Plan Focus Puppy Large Breed Chicken & Rice Formula
	Purina Pro Plan Focus Adult Sensitive Skin & Stomach Salmon & Rice Formula
	Purina Pro Plan Focus Small Bites Lamb & Rice Formula
	Purina Pro Plan Focus Adult Small Breed Formula Chicken and rice
	Purina Pro Plan Sport Active All Life Stages 26/16 Formula
	Purina Pro Plan Sport Performance 30/20 Salmon & Rice Formula
	Purina Pro Plan Sport All Life Stage Performance 30/20 Formula Chicken
	Purina Pro Plan Veterinary Diets HA Hydrolyzed Canine Formula (Vegetarian)
	Royal Canin Veterinary Care Nutrition Weight Control
	Royal Canin Golden Retriever Adult
	Royal Canin Miniature Schnauzer Adult
	Royal Canin Golden Retriever Puppy
	Royal Canin Satiety Support Weight Management
	Royal Canin Urinary SO
	Member's Mark Exceed DryDog Food, Chicken & Rice
	SportMix High Energy 26/18
	Victor Hi Pro Plus Active 88-2-10 Dog & Puppy
	Victor Performance Adult Maintenance with Glucosamine and Condroitin
	Victor Professional All Life Stages
