# Supplementary material for: Effect of type of diet on blood and plasma taurine concentrations, cardiac biomarkers, and echocardiograms in 4 dog breeds
Source: J Vet Intern Med. 2021 Feb 27;35(2):771–9. doi: 10.1111/jvim.16075 (PMC7995416; doi:10.1111/jvim.16075)
Supplement: Supplementary file 2 — TABLE S2 Diets with FDA‐listed ingredients of concern (peas, lentils, or potatoes) in the top 10 ingredients (FDA‐PLP) and diets without FDA‐listed ingredients of concern in the top 10 ingredients (NoFDA‐PLP) that were fed to enrolled dogs [file JVIM-35-771-s001.pdf]

Table S2. Diets with FDA-listed ingredients of concern (peas, lentils, or potatoes) in the top 10 ingredients (FDA-PLP) and diets without FDA-listed ingredients of concern in the top 10 ingredients (NoFDA-PLP) that were fed to enrolled dogs.

**FDA-PLP diets**	**NoFDA-PLP diets**
4Health Grain Free Chicken & Vegetables Formula Adult	4health Original Chicken & Rice Formula Adult
Acana Heritage Free-Run Poultry Formula	4health Original Performance
Acana Light and Fit Formula	4health Original Small Bites Formula Adult
Acana Meadowland	Actr1um Holistic
Aldi Pure Being Grain Free Salmon and Sweet Potato	Avoderm Adult Chicken Meal & Brown Rice Formula
American Journey Active Life Formula Large Breed Chicken, Brown Rice and Vegetables	Bil-Jac Adult Select Formula
Earthborn Holistic Primitive Natural	Diamond Naturals All Life Stages Dog Chicken & Rice Formula
Fromm Chicken A La Veg Recipe	Eukanuba Adult Small Bites Chicken Dry
Fromm Duck A La Veg Recipe	Evanger's Chicken and Brown Rice Recipe
Fromm Salmon Tunalini Recipe	Farmina N & D Ancestral Grain Chicken & Pomegranate Medium & Maxi Adult Dry
Halo Holistic Wild Salmon and Whitefish Recipe Adult	Fromm Adult Gold Dry
Hill's Science Diet Digestive Care i/d	Fromm Pork & Applesauce Formula
Honest Kitchen Zeal Grain Free Fish Recipe Dehydrated	Hills Science Diet Digestive Care i/d Low Fat
Kirkland Signature Adult Formula Chicken, Rice, & Vegetable	Iams ProActive Health Adult Minichunks
Merrick Grain Free Real Duck and Sweet Potato Recipe	Instinct Be Natural Real Chicken & Brown Rice Recipe
Merrick Grain Free Real Lamb and Sweet Potato Recipe	Nutrisource Adult Chicken & Rice Recipe
Natures Recipe Grain Free Chicken, Sweet Potato, & Pumpkin Recipe	Nutro Ultra Chicken/Lamb/Salmon Small Breed Weight Management
Nutrisource Pure Vita Salmon & Peas Entrée	Orijen Original Adult
Nutro Wholesome Essentials Large Breed Adult Pasture-Fed Lamb & Rice Recipe	Purina Beneful Healthy Puppy Dry Dog Food with Farm-Raised Chicken
Purina Pro Plan Focus Large Breed Chicken & Rice Formula	Purina One Smartblend Digestive Health Formula with Real Chicken
Redford Naturals Limited Ingredient Diet Grain Free Large Breed Lamb & Sweet Potato Recipe Adult	Purina One Smartblend Chicken & Rice Formula
Member's Mark Exceed DryDog Food, Chicken & Rice	Purina One Smartblend Lamb & Rice Formula
Simply Nourish Limited Ingredient Diet Salmon & Sweet Potato Recipe	Purina Pro Plan Savor Shredded Adult Beef & Rice Formula

Taste of the Wild High Prairie Canine Recipe with Roasted Bison and Roasted Venison	Purina Pro Plan Bright Mind Chicken & Rice Formula
Taste of the Wild Southwest Canyon Canine Recipe with Wild Boar	Purina Pro Plan Savor Shredded Blend Chicken and Rice Savor Formula
Taste of the Wild Pacific Stream Canine Recipe with Smoked Salmon	Purina ProPlan Focus Large Breed Chicken and rice
Taste of the Wild Pine Forest Canine Recipe with Venison & Legumes	Purina Pro Plan Focus Adult Sensitive Skin & Stomach Salmon & Rice Formula
Wellness Core Grain Free	Purina Pro Plan Focus Small Bites Lamb & Rice Formula
Zignature Salmon Limited Ingredient Formula	Purina Pro Plan Focus Adult Small Breed Formula Chicken and rice
	Purina Pro Plan Sport All Life Stages Active 26/16 Formula
	Purina Pro Plan Sport Performance 30/20 Salmon & Rice Formula
	Purina Pro Plan Sport All Life Stage Performance 30/20 Formula Chicken
	Purina ProPlan Veterinary Diets HA Hydrolyzed Canine Formula (Vegetarian)
	Royal Canin Veterinary Care Nutrition Weight Control
	Royal Canin Golden Retriever Adult
	Royal Canin Miniature Schnauzer Adult
	Royal Canin Golden Retriever Puppy
	Royal Canin Satiety Support Weight Management
	Royal Canin Urinary SO
	SportMix High Energy 26/18
	Victor Hi Pro Plus Active 88-2-10 Dog & Puppy
	Victor Performance Adult Maintenance with Glucosamine and Condroitin
	Victor Professional All Life Stages
